# Editorial: The Use of Biomaterials With Stem and Precursor Cells in Diseases of the Central Nervous System; A Step to Clinical Trials

**DOI:** 10.3389/fneur.2021.654890

**Published:** 2021-03-30

**Authors:** Rodrigo Ramos-Zúñiga, Hugo Guerrero-Cázares, Ulises Gómez-Pinedo, Jorge Matias-Guiu

**Affiliations:** ^1^Department of Neuroscience, University Center for Health Sciences (CUCS), University of Guadalajara, Guadalajara, Mexico; ^2^Department of Neurosurgery, Cancer Biology, and Neuroscience, Mayo Clinic Rochester, Rochester, MN, United States; ^3^Laboratory of Neurobiology, Hospital Clinico San Carlos, Health Research Institute San Carlos (IdISCC), Madrid, Spain; ^4^Department of Neurology and Laboratory of Neurobiology, Hospital Clinico San Carlos, Health Research Institute San Carlos (IdISCC), Universidad Complutense de Madrid, Madrid, Spain

**Keywords:** stem cells, biomaterials, cell therapy, clinical trials, experimental models - animal models, glioma, spinal cord injury, Alzheimer disease

With a participation of 12 articles, the Research Topic “The use of Biomaterials with Stem and Precursor Cells in Diseases of the Central Nervous System; A Step to Clinical Trials,” has had an impact that demonstrates the relevance and interest of translational research in scientific readers.

The link between the basic experimental scenario and the decision making process in the clinic is a highly demanding condition for public health and it strengthens the collaboration links between basic and applied research. With basic topics related to unique and combined molecular strategies and their diverse potential applications, this topic forms a mosaic of possibilities of technological development and therapeutic strategies in the short term.

Spinal cord injury, bioactive implants, scaffolds for tissue regeneration and proposals for chronic degenerative diseases such as Alzheimer's, cancer, and stroke, are evaluated with high scientific and ethical rigor. Their results promise new routes in the therapeutic possibilities from molecular and genetic concepts. These reports explore the handling of precursor cells and biomaterials, both as scaffolds and as vehicles of different bioactive factors with regenerative potential.

The evaluation of the state of the art in the use of biomaterials and stem and precursor cells is a crucial point in order to establish the goals and objectives in addressing new scientific and clinical challenges. At this point, Ojeda-Hernandez et al., include a detailed and extensive review on the biomedical applications of chitosan and its derivatives in the Central Nerve System (CNS). Several research groups have described the use of chitosan as a versatile biomaterial in the development of therapeutic strategies. The work presented by Lara-Velazquez et al., describes multiple studies highlighting the advantages and challenges of chitosan-based gene and drug delivery systems (nanocapsules, nanospheres, solid-gel formulations, nanoemulsions, microshperes, and micelles) for the treatment of brain tumors. The work presented by Ruiz-García, Alvarado-Estrada, Krishnan et al., addresses potential and newest developments in the area of bioengineering and cell therapy. It is an exquisite work, exploring state of the art approaches in the therapy of glioma. In recent years, the use of mesenchymal stem cells have taken a leading role in the development of therapeutic strategies in various pathologies of the nervous system. Hmadcha et al., describes the controversial role of the Mesenchymal Stem Cells (MSCs) in contributing to cancer pathogenesis mainly because the differentiation into the cancer associated fibroblast and also in cancer suppressing because the immunomodulation effect. MSC-based anti-cancer agent delivery systems were also described as a new potential application of MSCs.

Another pathology where the use of biomaterials preloaded with stem cells has been proposed to promote neural restoration is stroke. Esteban-Garcia et al., describe in their review the use of biomaterials for neurorestauration after ischemic stroke. One of the most common strategies to target this problem is biomaterials combined with cellular therapy. The authors address the limitations and consequences that originate after stroke, the endogenous repair mechanisms and the critical keys that can contribute to a successful therapy using biomaterials and stem cells.

A clear example of a topic for both basic and translational research is the review presented by Purvis et al., the authors describe the neurogenic potential of the adult brain and the capacity of neurogenic niches to serve as a potential source of cells that facilitate neuronal replacement injured or damaged regions of the brain. They show different strategies that are being studied in order to promote neuronal replacement from endogenous neural stem cells; cover different aspects from pharmacological approaches using neurotophic factors-loaded biomaterials. The work continues with a description of tissue engineered cellular scaffolds that allow neuroblast toward migration from neurogenic niches damaged brain areas. They propose the use of this particular technology for the development of therapies to treat several types of neurological disorders as well as for the treatment of traumatic brain injuries.

A new approach to the use of biomaterials is the design and *in vitro* three-dimensional (3D) modeling of neurodegenerative diseases such as Alzheimer's, Hernández-Sapiéns et al., in their research look for a novel Alzheimer disease study model. Similarly, Gazarian et al., for the first time, report the expression of tau protein and mRNA in Dental Pulp Stem Cells (DPSCs) in an *in vitro* model, this model is effective to study the mechanisms that promote tau aggregation and neurofibrillary tangles (NFT's) formation. Both reports manage to generate a valid and useful *in vitro* model useful in the area of personalized medicine. Along the same lines and with a similar methodological approach, Ruiz-Garcia, Alvarado-Estrada, Schiapparelli et al., give a comprehensive view of the multiple aspects of the glioma microenvironment which are fundamental for supporting tumor growth and phenotype, further describing how researchers have taken into account all these information for setting up 3D models able to recapitulate all these tumor aspects.

A further step in the advancement of preclinical trials is the development of experimental preclinical models that simulate pathologies such as spinal cord injury. Buzoianu-Anguiano et al., in their study, used predegenerate nerve scaffolding and bone marrow cell transplantation for spinal cord injury in a chronic complete transection model in rats. They describe beneficial effects with respect to axonal regeneration in the combined treatment groups, and attribute to this observation the slight motor functional improvements observed. In a similar model, Rodríguez-Barrera et al., investigate the impact of the combined strategy to improve morphological and functional recovery in rats with chronic spinal cord injury, addressing the use of stem cells, immunization strategies with neural peptides and scar removal.

Finally, an example of the convergence of basic research and its transfer to the clinic is the work presented by Ramos-Zúñiga et al., which addresses in the description of a clinical case of the surgical treatment to seal the skull floor. In this article, he reported the first case with the use of a novel bilaminar chitosan scaffold that can potentially be used in seal skull floor repair, primarily targeting the bony part of this structure.

We are confident that the path traced by this topic in biomedical research, can generate more projects and interest in the scientific community, from the synergy of the different domains of science, for the benefit of the population in the short term.

## Author Contributions

RR-Z, HG-C, UG-P, and JM-G: manuscript drafting and critical review of the manuscript. All authors contributed to the article and approved the submitted version.

## Conflict of Interest

The authors declare that the research was conducted in the absence of any commercial or financial relationships that could be construed as a potential conflict of interest.

